# Mechanical properties during healing of Achilles tendon ruptures to predict final outcome: A pilot Roentgen stereophotogrammetric analysis in 10 patients

**DOI:** 10.1186/1471-2474-8-116

**Published:** 2007-11-26

**Authors:** Thorsten Schepull, Joanna Kvist, Christer Andersson, Per Aspenberg

**Affiliations:** 1 Section for Orthopaedics and Sports Medicine, Department of Neurosciences and Locomotion, Faculty of Health Sciences, SE 581 85 Linköping, Sweden; 2 Section for Physiotherapy, Department of Medical and Health Sciences, 581 85 Linköping, Sweden

## Abstract

**Background:**

There are presently few methods described for in vivo monitoring of the mechanics of healing human tendon ruptures, and no methods for prediction of clinical outcome. We tested if Roentgen stereophotogrammetric analysis (RSA) can be used to follow the restoration of mechanical properties during healing of ruptured Achilles tendons, and if early measurements can predict clinical results.

**Methods:**

Achilles tendon repair was studied with RSA in 10 patients with a total rupture. Tantalum beads were implanted in conjunction with surgical repair. The patients were evaluated at 6, 12 and 18 weeks, and after 1 year. RSA was performed with two different mechanical loadings, and the strain induced by increasing load was measured. The transverse area was determined by ultrasound. CT scan at 12 weeks confirmed that the tantalum beads were located within the tendons. Functional testing was done after 1 year. A heel raise index was chosen as primary clinical outcome variable.

**Results:**

The strain was median 0.90, 0.32 and 0.14 percent per 100 N tendon force at 6 weeks, 18 weeks and one year respectively. The error of measurement was 0.04 percent units at 18 weeks. There was a large variation between patients, which appears to reflect biological variation. From 6 to 18 weeks, there was a negative correlation between increase in transverse area and increase in material properties, suggesting that healing is regulated at the organ level, to maximize stiffness. Modulus of elasticity during this time correlated with a heel raise index at one year (Rho = 0.76; p = 0.02).

**Conclusion:**

We conclude that the RSA method might have potential for comparing different treatments of Achilles tendon ruptures.

## Background

It is impossible to know when an individual patient with a tendon injury can first be recommended load-bearing, and how much load to start with. Radiographs don't help. Because there is no available information about the stage of repair in the individual patient, the post surgical treatment has to rely on rules of thumb, based on rerupture frequencies in the past [1,2]. Moreover, due to the lack of mechanical evaluation methods, it is hard to perform systematic study of different treatments to improve repair, such as with drugs [3-6] or controlled loading [3-6]. A method providing a measure for the properties of the healing tendon at a given time point would therefore be beneficial in the emerging research on tendon repair stimulation, and also in individual cases.

Probably, the final result of healing depends on early factors, such as type of injury, surgery and individual repair capacity, and late factors, such as different rehabilitation programs and the patient's motivation for training. An early measurement, describing the mechanical properties of the tendon callus might have a better sensitivity to early differences, such as surgical methods, and could also provide a base-line for studies of different rehabilitation protocols.

Tendon healing comprises formation of a new tendineous tissue [7] that restores continuum between the severed stumps, much like callus formation in bone healing. Strength, stiffness, resistance to creep and other mechanical properties improve continuously during repair, but little is known about what drives this process. Because measurement of mechanical properties involves measuring deformation for a known load, and roentgen-stereometric analysis (RSA) [8] can measure deformation in vivo in a non-destructive way, it seemed to be a practical method. RSA systems are commercially available [9] and commonly used in orthopaedic research clinics in Europe for bone implant stability studies. For RSA, small tantalum beads are inserted via a needle into the tissues. Simultaneous x-ray expositions in two planes inside a calibration cage then allow a description of the beads' position and position changes in space with a measuring error typically around 0.1 mm.

Thus, we employed RSA in order to describe the repair process for the ruptured Achilles tendon in mechanical terms. These results were then correlated to the functional outcome one year after the tendon rupture.

## Methods

The study was approved by the regional Ethics committee. All patients between 18 and 60 years of age, who presented an Achilles tendon rupture at the emergency department, were asked to participate after verbal and written information. 10 consecutive patients, having the required age, all agreed to participate between February and April 2004. The age was mean 39 years (range 29 – 48). There were 2 women. All were recreational athletes without previous Achilles tendon problems and had sustained their rupture during sports or sports-like activities, and all had consented to operative treatment.

All patients were operated on within 2 days after injury. They were operated in local anaesthesia using carbocaine with adrenaline and we used a conventional open technique with a dorso-medial approach. The distal and proximal tendon stubs were primarily adapted using one resorbable suture (Vicryl size 1) according to the single-loop Kessler technique and no further sutures. Then, with a special injection needle, 2 tantalum beads with a diameter of 0.8 mm were placed in the distal part of the Achilles tendon and 2 beads were placed in the proximal stub. 2 mL of fibrinogen-glue were injected into the rupture and around the fibres of the stubs. The sites where the tantalum beads had been inserted were covered by fibrin-glue. After suture of the paratenon and the skin, a short leg cast was applied with the ankle in equinus position. After 3 weeks the cast was exchanged to one in neutral position, for another 3 weeks. Full weight-bearing was allowed.

After 6 weeks the cast was removed and the patients were provided with shoes with an elevation of the heel of about 2 cm, that they were supposed to use for another 6 weeks. The patients started a standardized training program with supervision of a physiotherapist who saw the patient every second week until 24 weeks after surgery. This was according to the hospital's previous routines. Full weight-bearing was allowed. The patients were allowed to go back to full activity, including sports, at approximately 5 months.

**Figure 1:**
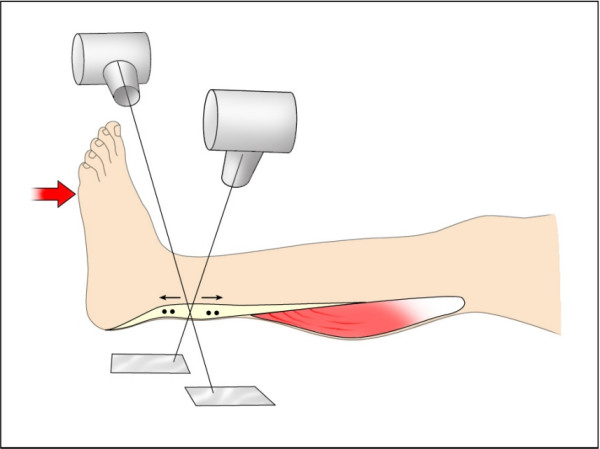
**RSA examinations**. RSA examinations performed at 6, 12 and 18 weeks and after one year with 25 and 200 N loading.

RSA is a method enabling accurate measurements of the distance between metal markers in 3 dimensions. It uses simultaneous x-ray exposures in two planes together with extra-corporal calibration markers in a cage [8]. A change in patient position from one examination to another (e.g. ankle flexion) does not influence the measurements if not the tissues are deformed. RSA examinations were performed at 6, 12 and 18 weeks after the operation. The first examination was performed within 48 hours after cast removal. A final RSA examination was done after one year, with a slightly different protocol (see below). For RSA, the patient sat with their leg horizontal and the foot placed in a frame, with 8 degrees plantar flexion. A pedal was then applied to the forefoot and adjusted to foot size. Via a system of wires, the pedal was then loaded with weights, and the patient was asked to resist the dorsiflecting force to keep the foot in position. The force applied to the pedal was 25 or 200 N. The 25 N force provided base-line values. It was chosen to be high enough to cause a reasonable relaxation of the dorsal flexor muscles. Four RSA radiographs were taken in a sequence with a loading of 25, 200, 25 and 200 N (Figure 1). Each load was applied for 15 s before x-ray exposition and then immediately removed. Care was taken to do the loadings (and expositions) with 3 min intervals. Between the second and third loadings the patient went down from the examination table and up again. Tendon force was calculated from pedal force. The pedal pivoted around an axis so that the force had a defined loading point in a lateral projection. Lever arms were calculated from ordinary lateral radiographs with the centre of the talar trochlea as pivot point (pedal point to trochlea centre and trochlea centre to centre of tendon).

At one year the examination consisted of a series of loadings for 15 s with 3 min intervals, but with no repeat loadings. The pedal forces were 25, 125, 225, 325, and 425 N.

**Figure 2:**
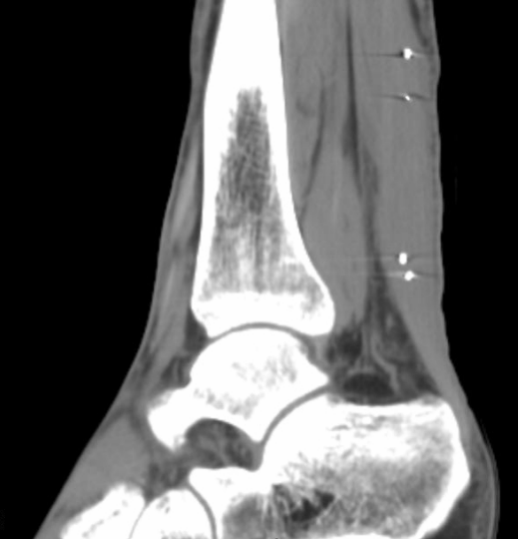
**CT**. CT after 12 weeks. Note the thick tendon callus. The two tantalum markers on each side of the healing rupture are enlarged due to artefacts.

All results (from 6, 12 and 18 weeks) are based on the second 25 and 200 N loadings, when not otherwise stated. The first pair of loadings was done for reproducibility estimation, and to serve as preconditioning loading. All results are based on the two beads close to the injury, when not otherwise stated. Strain per force values were adjusted to a calculated tendon force of 100 N, assuming linear relationships. Transverse area (at mid-distance from the proximal to the distal markers) was measured using ultrasound in conjunction with the RSA-examination after 6, 12 and 18 weeks and after one year. All measurements were performed by one experienced radiologist. A CT examination was done at 12 weeks, to confirm tantalum bead position (Figure 2). The RSA analysis used the UmRSA 4.1 system. Simultaneous exposures were done, using a calibration plate designed for RSA of the hip. Rigid bodies were not calculated. We used the software to calculate distances between single beads. Only the distance between the two beads close to the injury and the two beads farther away from the injury were measured (the difference between these two distances was also used to estimate strain in the uninjured region).

### Functional outcomes

Measurements of functional outcome were performed one year after the operation. All patients were asked about pain, weakness, limp, sensitivity to changes in weather, their ability to return to their previous level of activity and satisfaction with the outcome. Passive range of motion in dorsal extension and plantar flexion was registered with a handhold goniometer. The circumference of the calf was measured ten centimetres distal to the tibial tubercle. Tenderness over the tendon was evaluated with a 100 mm visual analogue scale (0 = no pain, 100 = worst imaginable pain). Gait pattern was evaluated with visual inspection for limping and toe off. The thickness of the tendon was measured with a slide calliper at the thickest place of the tendon. Muscle performance was evaluated with a number of tests: (1) maximal number of single limb toe raises (cadence 30 raises per minute, high of the heal should be minimum 5 cm from the floor), (2) maximal height for one single limb toe raise, (3) maximal time the patient could stay in a single limb toe raised position (cut off at 60 seconds), (4) isokinetic concentric peak torque for plantar flexion, tested on a Biodex machine at 30 degrees per second, (5) one leg jump for length, measured in cm and (6) one leg vertical jump registered on a Kistler force plate and measured in vertical height (vertical height = 9.8067 × (time in the air/2)^2^). Heel raise test has been recommended for evaluating calf muscle function [10]. The number of heel raises a person can perform is dependent on the height of the heel raise. Therefore, we created a heel raise index, defined as the number of heel raises the patient could do, multiplied with heel raise height as percentage of the other side. A modified Therman score was used to summarize objective and subjective parameters [11]. The Thermal score was assigned by the measures of range of motion, calf circumference, toe rises, Thompson test, isokinetic strength, pain, subjective weakness, return to previous level of activity, sensitivity to changes in weather and satisfaction with the outcome. The outcome has been categorized as excellent (90–100 points), good (80–89 points), fair (70–79 points) and poor (60–69 points).

### Statistics

The description of the evolution of mechanical properties involves correlations as described in the text. Normally, nonparametric statistics was used, but when residuals appeared normally distributed, a parametric correlation was sometimes used.

For testing the hypothesis that mechanics could predict functional outcome, the primary functional outcome variable was the heel raise index. This was chosen before any correlations with biomechanical or other measurements had been calculated. The mechanical variables were strain per force, modulus and transverse area. These were compared with the heel raise index. We used Spearman's (non-parametric) rank correlation, because of the low numbers and a clear outlier. In order to avoid multiple testing of dependent data, we produced one average value for each mechanical variable at 6, 12 and 18 weeks postoperative, and correlated them to the heel raise index. Thereafter, we proceeded to evaluate at the different time points one by one. Other comparisons were also done, but these should be regarded as descriptive, and p values are reported for information, but are not used to draw conclusions, because they are not corrected for possible mass significance.

## Results

### Mechanical description of tendon repair

There were no complications or problems that could be related to surgery or to the tantalum beads. There was no pain during testing. At the 6-weeks examination, 2 patients were unable to resist the 200 N weight, due to a feeling of weakness, and were tested with a 125 N weight instead. At the later examinations, there were no such problems.

### Mechanical estimates of healing between 6 and 18 weeks

**Figure 3:**
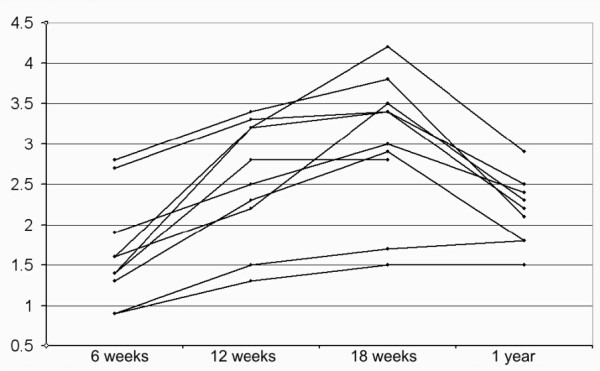
**Transverse area**. Development of transverse area (cm^2^)over time.

The transverse area increased in all patients from 6 to 12 weeks, and in all but one from 12 to 18 weeks (Wilcoxon signed rank test p = 0.005 and p = 0.008 respectively). The case with no increase remained constant (Figure 3). On average, the transverse area almost doubled from 6 to 18 weeks (Table 1). For each examination time, there was no correlation between transverse area and strain per force (r^2 ^= 0.00, 0.03 and 0.00 for 6, 12 and 18 weeks).

**Table 1:** Mechanical properties of healing tendons. Strain at 100 N for one year is calculated from regression line from all measurements. (Units: cm^2^, MPa, N/0.1 mm, % per 100 N)

	6 weeks				12 weeks				18 weeks				1 year			
	min	med	max	mean	min	med	max	mean	min	med	max	mean	min	med	max	mean

Transv area	0.9	1.5	2.8	1.7	1.3	2.7	3.4	2.6	1.5	3.2	4.2	3.0	1.8	2.3	2.9	2.3
Modulus	28	85	130	76	56	95	340	130	60	110	440	170	240	310	1000	410
Stiffness	11	21	36	22	19	43	103	55	29	63	207	81	68	134	319	143
Strain/force	0.55	0.90	1.47	0.97	0.16	0.40	0.78	0.41	0.09	0.32	0.46	0.27	0.04	0.14	0.20	0.14

Because the beads were inserted in the grossly uninjured tendon parts, the tissue between the beads contains both remnants of normal tendon and a regenerative site. Dependent on bead placement there could be a variation in the amount of grossly normal tendon included in the strain measurement. In order to see whether this could influence the results, we therefore compared strain per force values using either the two beads close to the injury or the two more distant ones, which include more "normal" tendon in the measurement. There was no correlation (at 6 weeks r^2 ^= 0.00; p = 0.4, at 18 weeks r^2 ^= 0.00; p = 0.9), indicating that strain per force was not influenced by the initial distance between the beads (on the other hand, stiffness values were influenced by this distance at 6 weeks; r^2 ^= 0.57; p = 0.01). This indicates that the mechanical properties of the tissue between the tantalum beads can be regarded as homogenous, which allows us to use strain per force as the directly measured relevant variable, and estimate the modulus of elasticity.

**Figure 4:**
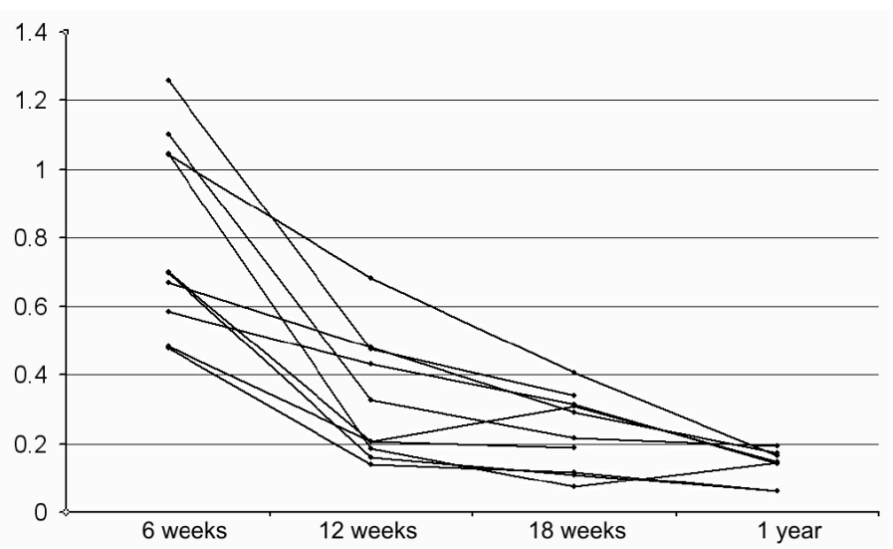
**Strain per force**. Development of strain per tendon force (%/100 N) over time.

The strain per force tended to have less variation than stiffness, and diminished by about half between each examination. (Wilcoxon signed rank test p = 0.005 and p = 0.007 respectively; Figure 4; Table 1). There was still a considerable variation at 18 weeks. To check that this does not represent methodological errors, the 18 weeks values were compared with the data from the beads distant from to the defect (r^2 ^= 0.95), and to the first (preconditioning) measurement (r^2 ^= 0.77).

**Figure 5:**
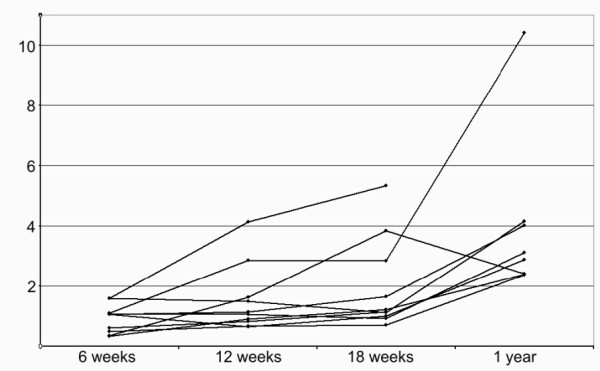
**Modulus**. Development of modulus (GPa) over time.

The modulus of elasticity showed a great variation. The mean value doubled from 6 to 18 weeks, but this was largely because a few tendons increased a lot. Thus, the median value was almost unchanged (Table 1), whereas in 3 cases the modulus reached much higher values than the others at 18 weeks (Figure 5). The stiffness showed a more clear increase with time, from median 21 N/mm at 6 weeks to median 63 N/mm at 18 weeks (Table 1). Three tendons had increased markedly more in stiffness at 18 weeks than the others.

**Figure 6:**
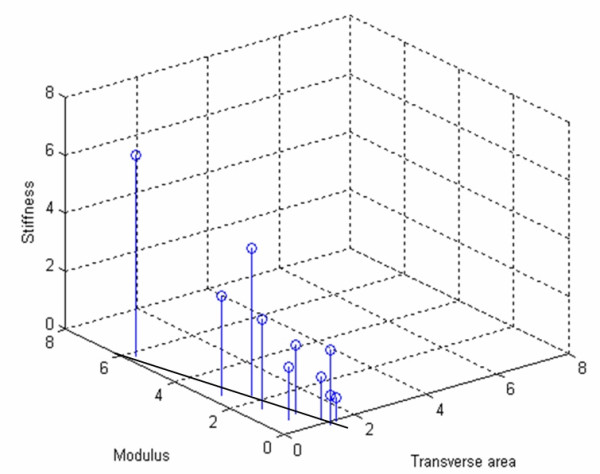
**Stiffness, transverse area and modulus**. Relation between the relative increases in stiffness, transverse area and modulus from 6 to 18 weeks (relative increases make the values independent of bead placement). Increase in stiffness is mostly caused by increasing modulus, but there is a negative correlation between increase in modulus and transverse area (symbolized by drawn line on bottom plane).

The relative increase in stiffness from 6 to 18 weeks did not correlate significantly to increase in transverse area (Spearman rank correlation, p = 0.6). The 3 tendons that increased most in stiffness had done so by increasing much in modulus, whereas they tended to increase very little in transverse area (Figure 6). Thus, there was a negative correlation between the increase in transverse area and increase in modulus (Spearman rank correlation, p = 0.008). Because this correlation could be caused by the fact that modulus was calculated from transverse area, we also correlated the area increase with increase in force per strain. This also yielded a negative correlation (Spearman rank correlation, p = 0.02), so it appears clear that the tendon callus either increases more in size and less in material properties, or vice versa (Figure 6).

### Preconditioning and error of measurement

Each examination comprised 2 pairs of measurements. The loading of the first pair (25 and 200 N) can be regarded as a preconditioning for the second one. In the 6 weeks examination, the tendons had a persisting elongation of 0.46 mm (0.8%) at the second 25 N load compared to the first one (sd 0.38; paired t-test p = 0.004). However, there was a similar persisting elongation at the second 200 N load compared to the first 200 N load (0.31 mm; p = 0.007). Therefore, there was no difference in tendon stiffness or strain between the measurements (elongation each time the load was increased from 25 to 200 N differed by mean 0.14 mm, sd 0.38; p = 0.27). The preconditioning effect remained at 18 weeks, when there was a 0.13 mm persisting elongation between the first and second 25 N loading (sd 0.19; p = 0.05). Error of measurement based on the difference in elongation of the 2 measurements of the first examination was 0.29 mm (sd for difference × 2^-0.5^). By comparing strain per 100 N at the 2 measurements of each examination, the error was 0.072 percent units at 6 weeks and 0.044 at 18 weeks. This corresponds to 8 and 16 percent of the mean values respectively.

### Effects on the undamaged tendon parts

The tendon was strained also in the uninjured region, i.e. the region between the two beads on each side of the injury. Therefore, the distance between the beads distant from the injury increased more upon loading than between the beads close to the injury (p = 0.0001). In 3 cases at the 6 weeks examination, the strain even appeared larger in the uninjured than in the injured region (uninjured region strain calculated from difference between distance increase between beads close to and distant from the injury). These latter calculations may however add several measuring errors and should be interpreted with care.

### Elongation over time

**Figure 7:**
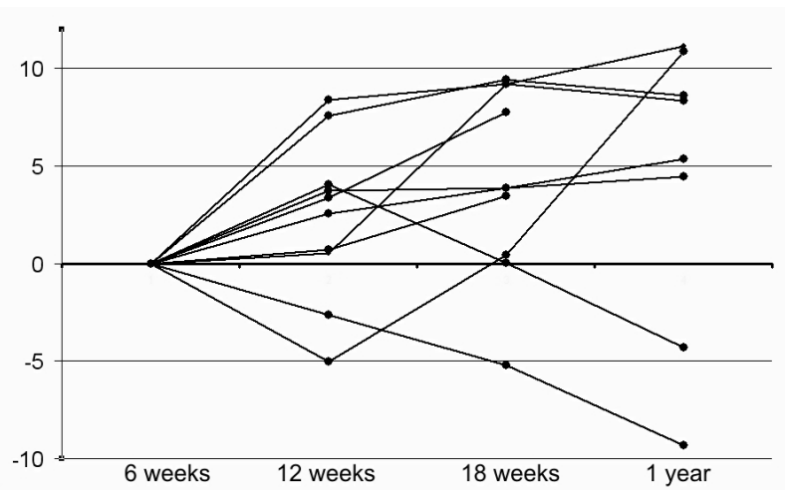
**Tendon length **. Change in tendon length (mm) over time.

The elongation of the tendons from 6 to 12 weeks (second measurement with 25 N weight, beads close to the injury) was median 3.0 (range -5.0 to 8.4) mm. Two tendons had shortened. There was less elongation from 12 to 18 weeks, median 1.6 (range -4.0 to 8.7) mm. Two tendons had shortened more than 2 mm. There was no median change in length between 18 weeks and one year, but 4 tendons had shortened, two of them over 4 mm. The change in length, from 6 weeks to one year was on average a lengthening of 3.5 mm, but ranged between a shortening of 9.3 to a lengthening of 11.2 mm (Figure 7). These figures appear to represent true changes in tendon length and not bead migration within the tendons, because when the measurements were repeated using the two beads distant from the injury, the results were very similar (r^2 ^= 0.97). Moreover, the elongation or shortening may have occurred also in the uninjured parts of the tendon, because in absolute terms, the change in distance between the two beads distant from the injury tended to be larger than between the other beads (Wilcoxon p = 0.09).

There was no correlation between tendon elongation over time and any mechanical property at one year.

### Tendon properties at 1 year

Two patients were lost from follow-up at 1 year, one of them did not want to participate and for the other, the examination failed due to technical problems. The transverse area had now decreased by one third from the 18-weeks examination (Wilcoxon p = 0.01). Strain per force was reduced by half, and modulus more than doubled in the same period (Wilcoxon p = 0.02 and p = 0.01 respectively). The tests with increasing load with 100 N increments from 25 to 425 N showed a linear relation between strain and load. In 6 of the 8 tendons r^2 ^was 0.93 or higher. Of the other patients, one had an r^2 ^of 0.82, and one of 0.68. In these two cases, technical mistakes during the examination may have occurred. Because this cannot be proven, they are not excluded from Table 1. However, without them the strain per force (calculated from the regression lines) would range between 0.12 and 0.20 percent/100 N. Modulus would range between 0.24 and 0.41 GPa.

No toe region could be observed on the curves, indicating that from 25 N load and higher the strain was mostly linear.

### Functional outcomes and correlations with mechanics

#### Descriptive statistics on measures of range of motion and muscle performance are presented in table 2

**Table 2:** Functional measurements at the one-year follow-up (mean (SD))

	Injured leg	Non-injured leg	Deficiency
Dorsal flexion (degrees)	35 (6)	38 (4)	9%
Plantar flexion (degrees)	41 (8)	46 (12)	12%
Calf circumference (cm)	37 (2)	38 (2)	4%
Tendon thickness (mm)	24 (2)	17 (2)	-34%
Max number of single toe raises	49 (43)	55 (29)	17%
Max height of a toe raise (cm)	9 (2)	11 (1)	18%
Isokinetic plantar flexion (Nm)	85 (15)	101 (13)	16%
One leg jump for length (cm)	117 (33)	127 (33)	8%
One leg vertical jump (cm)	25 (1)	30 (1)	9%

Six patients had not returned to their previous level of activity, because of fear of a new injury (n = 3), low functional ability (n = 1) and lack of time (n = 2). Three patients complained on tenderness over the tendon (25 mm on the VAS) approximately 12 cm from the heel. Visual gait analysis revealed normal gait pattern in all patients. The mean Therman score was 78. One patient had excellent results, 4 patients had good results, 3 patients had fair results and two patients had poor results. Tendon thickness, measured with a slide calliper, did not correlate with the tendon transverse area as measured with ultrasound (rho = 0.32, p = 0.44).

#### Correlation between tendon mechanical properties at one year and functional outcome

The heel raise index correlated with Therman score (Table 3). The modulus had a high and significant correlation with heel raise index. Although the heel raise index appeared to correlate with strain per force, this was not significant. Transverse area showed no correlation. The Therman and FIL scores correlated with the mechanical variables in a way similar to the heel raise index (Table 4).

**Table 3:** Non-parametric correlations between functional outcome variables at one year. N = 10. The FIL score was inverted

		Rho	p
Heel raise index	Therman score	0.95	0.007
Heel raise index	FIL score	0.62	0.08

**Table 4:** Correlations between functional outcome and one-year mechanical properties. N = 8. The FIL score was inverted. Strain per force has been inverted to become specific stiffness. Primary comparisons in bold

		Rho	p
Specific stiffness	Heel raise index	**0.57**	**0.13**
Specific stiffness	Therman score	0.62	0.10
Specific stiffness	FIL score	0.88	0.03
Modulus	Heel raise index	**0.88**	**0.02**
Modulus	Therman score	0.76	0.04
Modulus	FIL score	0.74	0.06
Transverse area	Heel raise index	**-0.29**	**0.42**
Transverse area	Therman score	-0.14	0.69
Transverse area	FIL score	0.33	0.39

One patient was an outlier. He had a completely normalized function, clearly better than the others. He also had a much stiffer tendon, with a higher modulus.

### Correlations between tendon mechanical properties during mobilization and heel raise index at one year

The average of the strain per force values at 6, 12 and 18 weeks after injury did not correlate with the heel raise index at one year, but the modulus showed a high and significant positive correlation. Similarly, the transverse area showed negative correlation, i.e. a thick callus appeared to predict an unfavourable outcome. When looking at each time point individually, the strain per force did not correlate with one-year heel raise index at any time point. However, the modulus did at 6 and 12 weeks, and the transverse area at 12 and 18 weeks (Table 5).

**Table 5:** Correlations between heel raise index and mechanical properties at 6, 12 and 18 weeks after injury. N = 10. Strain per force has been inverted to become specific stiffness. Primary comparisons in bold

		Rho	p
Specific stiffness	Mean 6, 12, 18	**0.29**	**0.39**
Modulus	Mean 6, 12, 18	**0.76**	**0.02**
Transverse area	Mean 6, 12, 18	**-0.71**	**0.03**
Specific stiffness	6	0.30	0.37
Specific stiffness	12	0.46	0.17
Specific stiffness	18	0.19	0.57
Modulus	6	0.66	0.05
Modulus	12	0.73	0.03
Modulus	18	0.59	0.08
Transverse area	6	-0.45	0.17
Transverse area	12	-0.69	0.04
Transverse area	18	-0.74	0.02

The change in tendon length from 6 weeks to one year did not influence heel raise index (rho -0.43, p = 0.26)

## Discussion

Functional results correlated with modulus and specific stiffness (inverted strain per force) one year after surgical treatment of Achilles tendon rupture. Early measurements of tendon modulus and transverse area, but not strain per force, could predict the functional one-year results. A larger number of patients would be needed to determine the strength of the prediction, but a parametric regression analysis suggests that the variance in modulus at plaster removal (6 weeks after injury) explains 45% of the variance in heel raise index (these data appear normally distributed). Although we have been looking for a large number of correlations, the significant p-values are hardly the result of random "mass-significance". First, we had chosen just one functional out-come variable (heel raise index), and correlated that to three interrelated biomechanical variables. After having shown significant correlations between the index and the average of the 6, 12 and 18 weeks examinations, it appears justified to proceed to look at the individual time points. The results from these time points are consistent.

We were first surprised to find that the modulus was better correlated with outcome than the strain per force was. The mechanical properties of the tendon as a whole are reflected by the strain per force. Modulus was calculated by relating strain per force to transverse area. Values for modulus therefore incorporate the errors of both these measurements, and still correlated better with outcome. The modulus is a material property, which should reflect the degree of tissue organization and maturity. The correlation with modulus suggests that tissue organization is more important to the patient than the amount of strain per force. The regenerated Achilles tendon needs not only be strong and stiff: it should also be able to function as a spring, with as complete recoil as possible. This requires that it should have a low degree of time-dependent deformation for a given load, i.e. a low hysteresis. It is likely that a higher degree of collagen organization (reflected by a high modulus) allows for a lower hysteresis and a better spring-like function. The correlation between modulus and out-come therefore suggests that the spring function is important.

We assume that Achilles tendon healing during the first 18 weeks mostly restores tensile strength and stiffness, and that there is a correlation between the two. The remodelling process that comes thereafter, and that lasts for years, might also tend to restore spring function. In the healthy Achilles tendon this function can transfer a large part of the mechanical energy from one single running step or hop to the next [12]. It requires that the tendon behaves in an elastic manner, i.e. the energy of the distension is recovered in the recoil. Moreover, the tendon "spring" must also have an eigenfrequency (resonance frequency), which is adapted to running movement (slightly higher than 1 Hz). The eigenfrequency of a spring is related to stiffness and coupled mass (frequency = (stiffness/mass)^0.5^). After one year of healing, the stiffness requirement regarding eigenfrequency should be met. The requirement of elastic behaviour is more likely to be problematic. The change in length from the first to the second pair of measurements in our study suggests a considerably viscoelastic behaviour, but we have not measured this at one year. Measurement of hysteresis is time-dependent and hardly possible with the RSA method. In vivo measurement of the mechanical properties of normal Achilles tendons have been done using ultrasound with the muscle-tendon junction or an inserted needle as marker [12-14]. This method allows real-time measurements, necessary to study hysteresis. The RSA method, on the other hand, is simple and practical, as many orthopaedic research departments already have the equipment.

The modulus at one year had barely reached half the level of normal Achilles tendons [12,14,15], except in one patient (who also happened to have an extraordinary good clinical result). Considering that the transverse area was increased 3 to 4 times compared to normal tendons, it appears that the Achilles tendon at one year should have a normal or higher stiffness. Our average strain per force values at one year correspond to a specific stiffness of 90 kN, to compare with 63 kN reported for normal Achilles tendons[16].

The large inter-individual variation in mechanics at 18 weeks appears truly biological, because the strain values were reproducible at the repeat examinations and similar if the tantalum beads closer or further away from the defect were used. At one year, there was a very good linear correlation between the 5 different loads applied and the corresponding strain, (except in 2 cases with reasonable correlation). Still, there was a large difference in strain per force between patients. This biological variation is hopeful, because it suggests a marginal for improvement, and corroborates the idea of using mechanical measurements as a prognostic variable.

We have used RSA equipment for our measurements, but the analysis is different from the standard RSA, because we could not use "rigid bodies" in the tendon stumps. Thus, there is no inherent control for accuracy in each measurement as in normal RSA. However, we are confident that no bead migration within the tendon substance has occurred during the 3 minutes between the different x-ray exposures of one examination. Bead migration during the weeks between examinations cannot be excluded, but the very high correlation between the distances between the two beads close to the injury and the two beads farther away speaks strongly against it.

We believe that specific stiffness (N/strain) and modulus are more relevant than stiffness (N/mm). If the ruptured tendon were two rigid stumps with a ductile tissue in between, the original distance between the marker beads inside the stumps would not influence lengthening in mm for a given load increase. If, on the other hand, the ductility were homogenous in the entire segment between the beads, lengthening (mm) would be influenced by bead position, but strain would not be influenced. This could be tested by comparing the pair of beads close to the rupture with the pair of beads distant from the rupture. We found that the original distance between the beads did not influence values for strain per force, whereas stiffness was influenced. This indicates mechanical homogeneity of the tendon segment between the beads and that strain per force is a better measure than stiffness. In healthy tendons, different fascicles may have different modulus [17], but at this stage of repair, a regenerating tendon appears histologically homogenous according to observations in animals [18]. Furthermore, we have observed in a series of repeated CT examinations that tendon thickening and callus formation occurs along the entire tendon, and also that radio-density is decreased along the entire callus, indicating a homogenous increase in water content (manuscript in preparation).

In order to elucidate the possible relation between strain per force and ultimate tensile strength, we went back to the data from a rat Achilles tendon transection study [18]. In this experimental set-up stiffness and specific stiffness are identical. The data showed that after 21 days of healing there was a clear correlation between stiffness and force at failure (control animals, n = 10, r^2 ^= 0.66, p = 0.003). This time point might correspond to about 12 weeks in man. Early in repair, day 8, the correlation was weaker (control animals, n = 46, r^2 ^= 0.27, p = 0.0002). Intact rat tendons (that ruptured in the tendon substance) also had a correlation between stiffness and force at failure (n = 23, r^2 ^= 0.53, p = 0.0001). These animal data support the assumption that clinical data for strain per force are related to tendon strength.

The changes in transverse area and modulus from 6 to 18 weeks might be of theoretical interest. The inverse relationship between increase in material properties and increase in dimensions suggests that the healing process is regulated by a mechanism that tries to obtain optimal stiffness, so that smaller increase in modulus is compensated by more growth in size and vice versa (Figure 6). Thus it seems that early healing is optimised on the organ level, since stiffness (and strain per force) are properties of the whole construct. In order to do this, the tendon cells must detect a construct-related factor, such as fluid flow, rather than cell deformation [19].

Most tendons elongated several millimetres during the studied time period, but some shortened. This has been demonstrated previously using inserted metal markers [20-22]. Changes in length might also have occurred during the first postoperative weeks, which we did not investigate. However, we found a continuing change in length also after 18 weeks. This may represent an adaptation to mechanical requirements. Not only the muscle, but also the tendon appears to have a considerable ability to compensate for errors in the restoration of tendon length at surgery. Interestingly, the change in length during the year did not influence the clinical results.

The prediction of out-come by the modulus at plaster removal could have two explanations. The individual patients could have different characteristics, i.e. some have a better ability for tendon regeneration than others, this phenomenon showing up early. However, a perhaps more likely explanation would be that early modulus reflects the operative result, i.e. the type of injury and the quality of the surgery. Under all circumstances it is clear that the end result is in part influenced by factors unrelated to training, because the predictive examination was done before training started.

## Conclusion

This feasibility study has few patients, and although significant correlations were found, they should be confirmed in a larger study before firm conclusions about predictive possibilities can be drawn. If it is confirmed that a quantitative examination can be done at plaster removal, this indicates that we may have a more sensitive method for comparing surgical methods before any difference is blurred by differences in training, socio-economics and personality. Moreover, it might become possible to compare training protocols, using the values at plaster removal as base-line, and thus eliminate some variation due to surgical or injury differences. A final possibility would be to use RSA for monitoring of postoperative training in selected cases. The present data are insufficient for any recommendations on how to do that. However, it is likely that patients with an extremely high strain per force, i.e. a mechanically inferior tendon construct, have a larger risk of re-rupture.

## Competing interests

The author(s) declare that they have no competing interests.

## Authors' contributions

TS participated in planning the study, operated most patients, made all RSA examinations, participated in data analysis and manuscript writing.

CA participated in planning the study, operated some patients and participated in manuscript writing.

JK planned and made all physiotherapeutic follow-up, participated in data analysis and manuscript writing.

PA planned the study, made most of data analysis and writing.

All authors have read and approved this final manuscript.

## Pre-publication history

The pre-publication history for this paper can be accessed here:


